# Supplementary material for: Stable ocean redox during the main phase of the Great Ordovician Biodiversification Event
Source: Commun Earth Environ. 2022 Sep 22;3(1):220. doi: 10.1038/s43247-022-00548-w (PMC9510202; doi:10.1038/s43247-022-00548-w)
Supplement: Supplementary file 2 — Supplementary Information [file 43247_2022_548_MOESM2_ESM.pdf]

SUPPLEMENTARY INFORMATION

Supplementary Discussion

Diagenesis

Carbonate diagenesis shifts $\delta^{238}U$ signatures towards higher values with an offset of 0.27±0.28 (2sd) relative to contemporaneous seawater[1]. Thus, the presented $\delta^{238}U$ results have the potential to be higher compared to the original seawater signatures. Nevertheless, here we evaluate the evolution of $\delta^{238}U$ trends throughout the Middle Ordovician and so obtaining the absolute $\delta^{238}U$ seawater values[2] is out of the scope of this work.

Cross-plots show no covariation between U isotopes and elemental ratios indicative of processes taking place during carbonate diagenesis (Mg/Ca for dolomitization; Sr/Ca for variations in the proportion of precursor aragonite versus calcite; Al/Ca for detrital input; and Mn/Sr for evaluating the influence of subsurface or diagenetic fluids that mostly are low in Sr and high in Mn) (Supplementary Fig. 1). Thus, we suggest that those diagenetic processes did not systematically influence the obtained $\delta^{238}U$ values. Mn/Sr ratios are high (>1), which traditionally have been interpreted as an indicator of diagenetic alteration. However, the lack of coupling between this ratio and $\delta^{238}U$ has previously been shown and recent work has concluded that Mn/Sr ratios do not faithfully determine the degree of diagenetic alteration[3]. Thus, we suggest this lack of covariation indicates the processes controlling Mn/Sr did not affect our $\delta^{238}U$ results.

Relationship between stratigraphy and geochemical and isotopic data

There is an apparent relationship between stratigraphic units, elemental ratios (U/Ca. Fig. 3; Mn/Sr. P/Ca and V/Ca Supplementary Fig. 2), and some of the $\delta^{238}U$ results. The Lanna and Holen limestones (excluding the Täljsten interval, Dapingian–Dw2. Fig. 3) are characterized by the lowest elemental ratios (particularly U/Ca < 0.01). and the Skövde limestone and overlying Gullhögen Fm. (Dw3; Fig. 3) are characterized by higher elemental ratios (U/Ca > 0.01; and also higher Mn/Sr, P/Ca and V/Ca; Supplementary Fig. 2) and the largest and most fluctuating $\delta^{238}U$ trend (from –0.78 to 0.16‰; 0.27 1sd). Similarly, the Täljsten interval within the Holen limestone shows a peak in P/Ca, Mo/Ca and U/Ca (Supplementary Fig. 2) in association with an abrupt swing in $\delta^{238}U$ values from –0.67 to –0.25‰ (Fig. 3). Thus, our $\delta^{238}U$ results and elevated U/Ca and Mo/Ca further suggest that the Täljsten interval was deposited under locally more reducing conditions[4], as U and Mo are enriched in sediments with increasing reducing conditions; e.g. more sulfidic pore fluids[5,6]. Similar elemental enrichments have recently been described in the same stratigraphic units from a section near the Kinnekulle drillcore (Hällekis Quarry, N 58°36.54232′, E 13°23.69535′), and they have been interpreted as likely due to the effects of important sea-level regressions changing sedimentation rates and early diagenetic redox potentials below the sediment-water interface (for more details see ref. [7].

SUPPLEMENTARY TABLE

Table S1. $\delta^{13}C$, $\delta^{238}U$ and elemental ratios of Kinnekulle-1 drillcore from Kinnekulle, Västergötland. Sweden. The lithological shift corresponding to the Täljsten within the Holen Limestone appears in *italic*.

Sample	Position (m)	$\delta^{13}C_{carb}$ (‰ V-PDB)	$\delta^{238}U$ (‰)	2se (‰)	Mg/Ca wt%/wt%	Sr/Ca wt%/wt%	Mn/Sr ppm/ppm	Al/Ca wt%/wt%	Fe/Ca wt%/wt%
Uli-2	0.3	-0.17	-0.22	0.05	0.012	0.0008	5.0	0.01	0.017
Uli-3	0.7	-0.10	-0.32	0.04	0.016	0.0008	4.0	0.01	0.019
Uli-6	2.3	-0.15	-0.51	0.13	0.015	0.0007	4.2	0.01	0.016
Uli-7	2.8	0.25	-0.20	0.07	0.018	0.0008	3.2	0.01	0.022
Uli-8	3.2	0.34	-0.39	0.08	0.011	0.0007	3.8	0.01	0.008
Uli-9	4.0	0.38	-0.39	0.04	0.013	0.0008	4.2	0.01	0.015
Uli-10	4.5	0.30	-0.27	0.05	0.012	0.0008	3.8	0.01	0.012
Uli-11	5.0	0.30	-0.33	0.04	0.011	0.0007	4.0	0.01	0.011
Uli-12	5.6	0.30	-0.27	0.04	0.012	0.0007	4.2	0.01	0.015
Uli-13	6.1	0.41	-0.36	0.04	0.012	0.0007	4.5	0.01	0.011
Uli-14	6.5	0.58	-0.25	0.05	0.010	0.0007	5.1	0.01	0.009
Uli-15	7.0	0.57	-0.22	0.04	0.010	0.0007	3.8	0.01	0.008
Uli-16	7.6	0.38	-0.28	0.13	0.012	0.0007	4.2	0.01	0.014
Uli-17	8.0	0.30	-0.33	0.06	0.012	0.0007	4.0	0.01	0.012
Uli-18	8.6	0.50	-0.41	0.04	0.010	0.0007	5.3	0.01	0.008
Uli-19	9.0	0.43	-0.48	0.05	0.011	0.0007	5.4	0.01	0.011
Uli-21	10.1	0.37	-0.38	0.05	0.010	0.0006	5.3	0.01	0.009
Uli-22	10.5	0.39	-0.44	0.06	0.011	0.0007	4.6	0.01	0.010
Uli-23	11.0	0.43	-0.41	0.04	0.010	0.0007	4.6	0.01	0.011
Uli-24	11.6	0.37	-0.40	0.05	0.010	0.0007	5.4	0.01	0.011
Uli-25	12.1	0.47	-0.53	0.04	0.008	0.0007	5.9	0.01	0.007
Uli-29	14.0	0.49	-0.33	0.04	0.010	0.0007	7.4	0.01	0.016
Uli-30	14.5	0.95	-0.34	0.03	0.009	0.0009	5.3	0.01	0.010
Uli-32	15.8	0.60	-0.51	0.05	0.010	0.0007	6.1	0.01	0.010
Uli-33	16.2	0.61	-0.34	0.03	0.009	0.0007	6.7	0.01	0.008
Uli-34	16.7	0.61	-0.40	0.04	0.008	0.0007	7.1	0.01	0.006
Uli-37	*18.4*	*0.79*	*-0.67*	*0.05*	*0.006*	*0.0008*	*9.5*	*0.00*	*0.004*
Uli-38	*18.9*	*0.73*	*-0.41*	*0.05*	*0.006*	*0.0008*	*8.6*	*0.00*	*0.006*
Uli-39	*19.1*	*0.69*	*-0.25*	*0.03*	*0.008*	*0.0008*	*6.6*	*0.00*	*0.007*
Uli-41	20.4	0.67	-0.46	0.08	0.009	0.0007	6.7	0.01	0.006
Uli-45	22.8	0.69	-0.30	0.04	0.009	0.0008	6.6	0.01	0.007
Uli-46	23.4	0.71	-0.25	0.03	0.010	0.0008	6.0	0.01	0.010
Uli-47	23.8	0.81	-0.23	0.06	0.010	0.0008	6.5	0.01	0.012
Uli-49	24.9	0.87	-0.31	0.06	0.010	0.0008	6.9	0.01	0.009
Uli-50	25.4	0.85	-0.16	0.04	0.010	0.0008	8.4	0.01	0.010
Uli-51	25.7	0.90	-0.14	0.04	0.010	0.0007	9.6	0.01	0.010
Uli-52	26.4	1.09	-0.27	0.03	0.010	0.0007	11.8	0.01	0.013
Uli-53	27.0	1.19	-0.23	0.03	0.012	0.0009	6.6	0.01	0.015
Uli-54	27.5	1.15	-0.49	0.10	0.012	0.0010	6.3	0.02	0.020
Uli-55	28.0	1.14	-0.36	0.04	0.013	0.0009	8.1	0.02	0.020
Uli-56	28.6	0.87	-0.40	0.03	0.012	0.0009	13.8	0.02	0.029
Uli-57	29.0	0.70	-0.47	0.04	0.015	0.0011	17.7	0.02	0.034
Uli-58	29.5	0.86	-0.41	0.04	0.012	0.0008	19.0	0.02	0.025
Uli-59	29.7	1.01	0.06	0.04	0.014	0.0008	14.4	0.01	0.025
Uli-60	30.2	0.56	-0.77	0.11	0.011	0.0011	12.7	0.01	0.033
Uli-61	30.8	0.80	-0.78	0.03	0.009	0.0009	16.5	0.01	0.027
Uli-62	31.3	0.79	-0.10	0.04	0.014	0.0011	10.9	0.01	0.032
Uli-63	31.8	0.61	-0.51	0.03	0.009	0.0010	13.3	0.01	0.027
Uli-64	32.3	0.58	-0.48	0.04	0.009	0.0009	16.7	0.01	0.025
Uli-67	34.0	0.69	-0.14	0.04	0.012	0.0010	22.7	0.01	0.038
Uli-68	34.4	0.55	-0.28	0.03	0.014	0.0011	23.5	0.02	0.054
Uli-69	35.0	0.95	-0.57	0.04	0.023	0.0016	11.5	0.04	0.078
Uli-70	35.5	0.84	-0.36	0.04	0.023	0.0015	16.3	0.04	0.075
Uli-71	36.1	0.36	-0.19	0.04	0.019	0.0014	18.5	0.03	0.069
Uli-72	36.8	0.73	0.03	0.06	0.014	0.0010	19.2	0.01	0.035
Uli-73	37.4	0.77	-0.07	0.03	0.012	0.0010	17.0	0.01	0.032
Uli-74	37.9	0.73	-0.19	0.03	0.012	0.0009	19.9	0.01	0.021
Uli-75	38.5	0.69	-0.27	0.04	0.012	0.0008	19.4	0.01	0.024
Uli-76	39.1	0.43	-0.03	0.04	0.013	0.0009	13.0	0.01	0.025
Uli-77	39.6	0.67	0.16	0.03	0.011	0.0008	13.6	0.01	0.018
Uli-78	40.2	0.63	-0.16	0.04	0.011	0.0008	12.2	0.01	0.021
Uli-79	40.7	0.44	0.01	0.04	0.011	0.0007	13.3	0.01	0.016

Table S1. (continuation)

Sample	P/Ca (ppm/wt.%)	V/Ca (ppm/wt.%)	Mo/Ca (ppm/wt.%)	U/Ca (ppm/wt.%)
Uli-2	0.001	0.14	0.0009	0.004
Uli-3	0.000	0.15	0.0004	0.003
Uli-6	0.000	0.11		0.003
Uli-7	0.001	0.16	0.0001	0.003
Uli-8	0.000	0.07		0.003
Uli-9	0.001	0.12		0.004
Uli-10	0.000	0.09		0.003
Uli-11	0.000	0.10	0.0014	0.003
Uli-12	0.000	0.14	0.0017	0.003
Uli-13	0.000	0.14	0.0016	0.003
Uli-14	0.000	0.10	0.0005	0.003
Uli-15	0.000	0.08	0.0007	0.003
Uli-16	0.001	0.12	0.0002	0.004
Uli-17	0.001	0.13	0.0013	0.004
Uli-18	0.000	0.10	0.0003	0.003
Uli-19	0.000	0.11	0.0004	0.004
Uli-21	0.000	0.11	0.0005	0.004
Uli-22	0.000	0.11	0.0011	0.003
Uli-23	0.001	0.12	0.0021	0.004
Uli-24	0.001	0.12	0.0023	0.004
Uli-25	0.000	0.10	0.0006	0.003
Uli-29	0.001	0.11	0.0004	0.003
Uli-30	0.001	0.07	0.0008	0.002
Uli-32	0.001	0.13		0.003
Uli-33	0.000	0.12		0.003
Uli-34	0.000	0.10		0.002
Uli-37	*0.006*	*0.05*	*0.0053*	*0.105*
Uli-38	*0.004*	*0.11*	*0.0034*	*0.083*
Uli-39	*0.006*	*0.11*	*0.0011*	*0.009*
Uli-41	0.000	0.07	0.0002	0.002
Uli-45	0.000	0.07	0.0024	0.002
Uli-46	0.001	0.09	0.0004	0.003
Uli-47	0.000	0.08	0.0001	0.003
Uli-49	0.001	0.09	0.0012	0.002
Uli-50	0.000	0.11	0.0037	0.003
Uli-51	0.000	0.09	0.0007	0.002
Uli-52	0.001	0.11	0.0008	0.003
Uli-53	0.001	0.13	0.0004	0.004
Uli-54	0.001	0.17	0.0009	0.007
Uli-55	0.002	0.18	0.0004	0.005
Uli-56	0.001	0.37		0.007
Uli-57	0.003	0.30	0.0006	0.016
Uli-58	0.001	0.23		0.007
Uli-59	0.003	0.64		0.146
Uli-60	0.002	0.44	0.0032	0.054
Uli-61	0.001	0.28	0.0026	0.052
Uli-62	0.001	0.22		0.005
Uli-63	0.001	0.31	0.0129	0.073
Uli-64	0.001	0.24	0.0001	0.022
Uli-67	0.001	0.36	0.0022	0.006
Uli-68	0.002	0.49	0.0026	0.015
Uli-69	0.005	0.75	0.0025	0.041
Uli-70	0.003	0.63	0.0037	0.033
Uli-71	0.003	0.64	0.0007	0.021
Uli-72	0.001	0.27	0.0008	0.013
Uli-73	0.001	0.25	0.0014	0.017
Uli-74	0.002	0.45	0.0007	0.101
Uli-75	0.001	0.28	0.0006	0.033
Uli-76	0.002	0.19	0.0002	0.011
Uli-77	0.001	0.16	0.0017	0.022
Uli-78	0.001	0.18	0.0027	0.005
Uli-79	0.001	0.13	0.0010	0.005

Table S2. Comparison of $\delta^{238}U$ data from the modern Bahamas carbonate platform[1,5,8], and interval I and interval II in the Kinnekulle-1 drillcore from Kinnekulle, Västergötland. Sweden. A) Observational $\delta^{238}U$ data. B) Statistical F-tests documenting distinct variances.

A)

Bahamas	Interval I	Interval II
0.01	-0.06	-0.67
0.01	-0.22	-0.41
0.1	-0.32	-0.25
0.11	-0.51	-0.46
0.09	-0.20	-0.30
0.09	-0.39	-0.25
-0.1	-0.39	-0.23
-0.31	-0.27	-0.31
-0.43	-0.33	-0.16
-0.45	-0.27	-0.14
-0.14	-0.36	-0.27
-0.37	-0.25	-0.23
-0.35	-0.22	-0.49
0.08	-0.28	-0.36
-0.2	-0.33	-0.40
-0.11	-0.41	-0.47
-0.03	-0.48	-0.41
-0.08	-0.38	0.06
-0.12	-0.44	-0.77
-0.23	-0.41	-0.78
-0.11	-0.40	-0.10
0.15	-0.53	-0.51
0.13		-0.48
-0.23		-0.14
-0.18		-0.28
-0.16		-0.57
-0.23		-0.36
-0.47		-0.19
-0.47		0.03
-0.02		-0.07
0.01		-0.19
-0.32		-0.27
-0.32		-0.03
-0.17		0.16
-0.18		-0.16
-0.1		0.01
-0.11		
-0.37		
-0.33		
-0.19		
-0.28		
-0.27		
-0.12		
-0.16		
-0.18		
0.02		
0.04		
-0.15		
-0.19		
0.02		
0.01		
-0.28		
-0.26		
-0.15		
-0.15		
-0.08		
-0.13		
-0.07		
-0.1		
-0.06		
-0.12		
-0.26		
-0.24		
0		
0.08		
0.07		
-0.01		

B)

Testing whether Bahamas has larger variance than Interval I

	**Bahamas**	**Interval I**
	0.01	-0.2237631
Mean	-0.1384018	-0.3588546
Variance	0.0170647	0.00884244
Observations	150	20
df	149	19
F	1.9298645	
P(F<=f) one-tail	0.04876569	
F Critical one-tail	1.92031972	
	0.01	-0.2237631

Interpretation:
The variances of Bahamas and Interval I data are distinct (CL = 95%)

Testing whether Interval II has larger variance than Bahamas

	**Interval II**	**Bahamas**
	-0.6673033	0.01
Mean	-0.2795871	-0.1384018
Variance	0.04627099	0.0170647
Observations	35	150
df	34	149
F	2.7115028	
P(F<=f) one-tail	1.9138E-05	
F Critical one-tail	1.50834628	

Interpretation:
The variances of Interval II and Bahamas data are distinct (CL > 99.99%)

-0.02<br>0.06<br>0.05<br>0.15<br>0.18<br>0.05<br>-0.17<br>-0.22<br>-0.27<br>-0.15<br>-0.01<br>0.02<br>-0.17<br>-0.17<br>-0.19<br>-0.18<br>-0.17<br>-0.01<br>-0.21<br>-0.19<br>0.02<br>-0.29<br>-0.25<br>-0.17<br>-0.23<br>-0.21<br>-0.13<br>-0.21<br>-0.16<br>-0.18<br>-0.1<br>-0.15<br>0.02<br>-0.21<br>-0.3<br>-0.25<br>-0.19<br>-0.12<br>-0.01<br>-0.19<br>-0.12<br>-0.15<br>-0.13<br>-0.16<br>0.03<br>0.07<br>0.14<br>-0.16<br>-0.13<br>-0.21<br>-0.19<br>-0.12<br>-0.23<br>-0.24<br>-0.16<br>-0.18<br>-0.18<br>-0.22<br>-0.17<br>-0.03<br>-0.06<br>-0.27<br>-0.19<br>-0.18<br>-0.20<br>-0.20<br>-0.20<br>-0.02<br>-0.22<br>-0.21<br>0.00<br>-0.30<br>-0.26<br>-0.19<br>-0.17<br>-0.23<br>-0.15<br>-0.23<br>-0.17<br>-0.20<br>-0.12<br>-0.17<br>0.00<br>-0.23	

SUPPLEMENTARY FIGURES

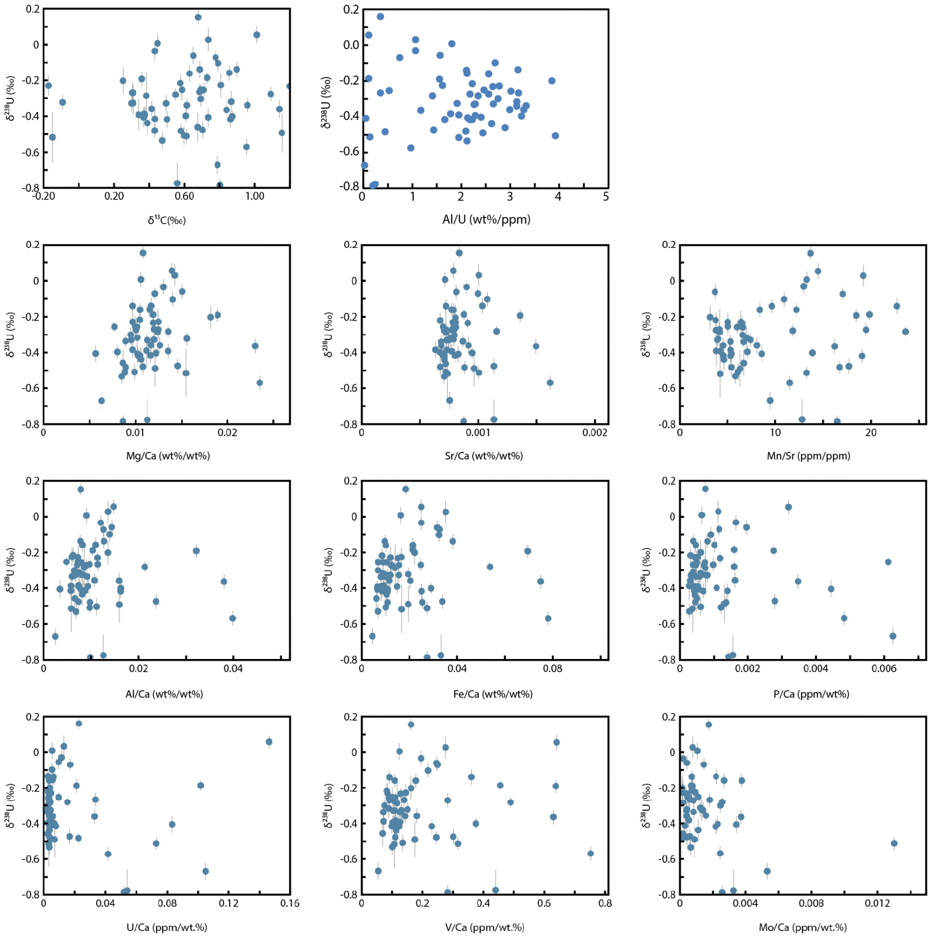

Supplementary Fig. 1. Cross-plots of $\delta^{238}U$ vs $\delta^{13}C$, Al/U, Mg/Ca, Sr/Ca, Mn/Sr, Al/Ca, Fe/Ca, P/Ca, U/Ca, V/Ca, and Mo/Ca for the Kinnekulle-1 drillcore. Error bars represent 2 s.e. analytical error for $\delta^{238}U$.

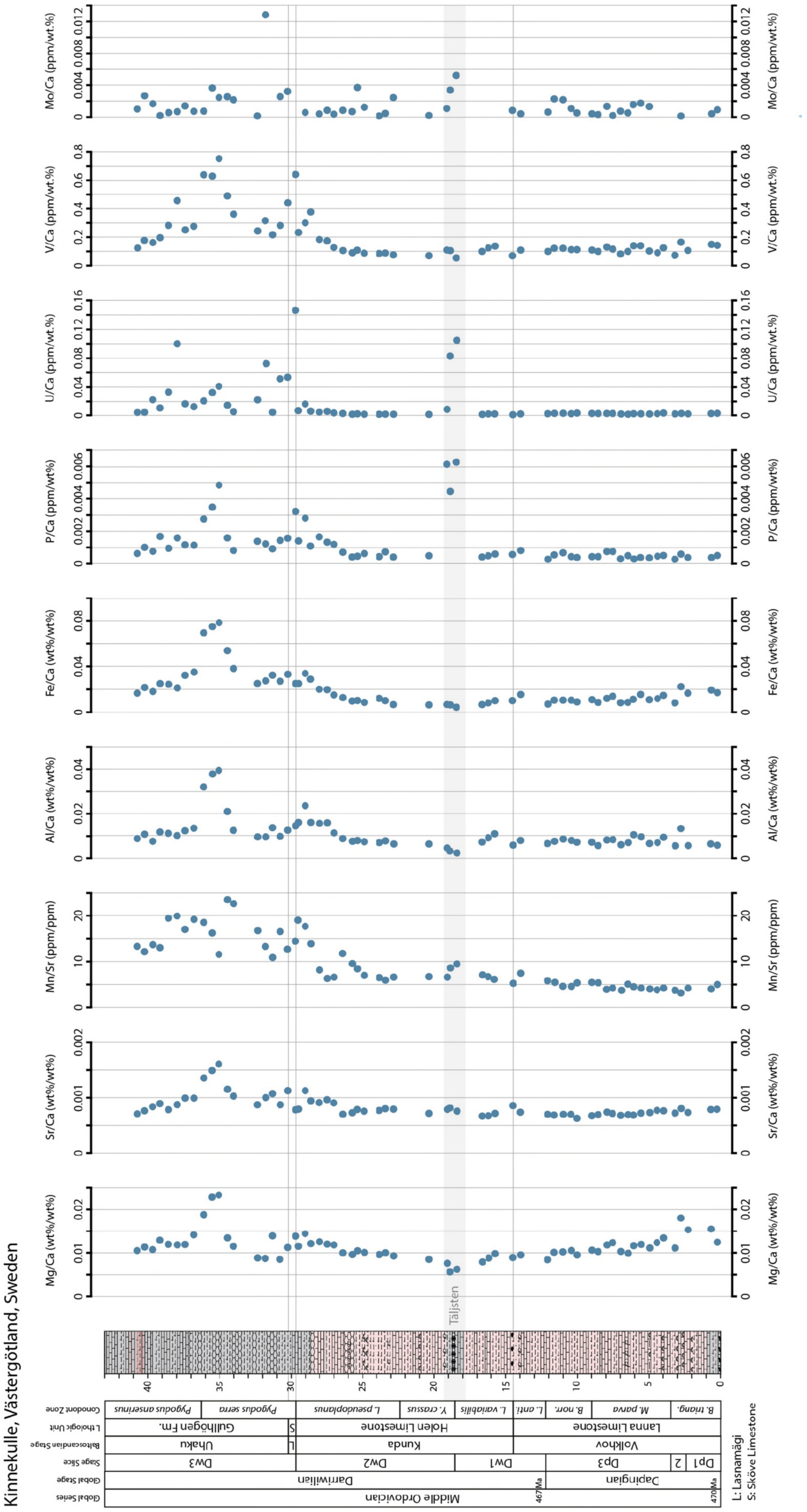

Supplementary Fig. 2. Elemental ratios through the Kinnekulle-1 drillcore. The grey area indicates the Täljsten. The grey lines represent unit boundaries.

REFERENCES SUPPLEMENTARY INFORMATION

1. Chen, X. *et al.* Diagenetic effects on uranium isotope fractionation in carbonate sediments from the Bahamas. *Geochimica et Cosmochimica Acta* **237**, 294–311 (2018).
2. del Rey, Á., Havsteen, J. C., Bizzarro, M. & Dahl, T. W. Untangling the diagenetic history of uranium isotopes in marine carbonates: A case study tracing the δ238U composition of late Silurian oceans using calcitic brachiopod shells. *Geochimica et Cosmochimica Acta* **287**, 93–110 (2020).
3. Higgins, J. A. *et al.* Mineralogy, early marine diagenesis, and the chemistry of shallow-water carbonate sediments. *Geochimica et Cosmochimica Acta* **220**, 512–534 (2018).
4. Eriksson, M. E. *et al.* Biotic dynamics and carbonate microfacies of the conspicuous Darriwilian (Middle Ordovician) 'Täljsten' interval, south-central Sweden. *Palaeogeography, Palaeoclimatology, Palaeoecology* **367–368**, 89–103 (2012).
5. Romaniello, S. J., Herrmann, A. D. & Anbar, A. D. Uranium concentrations and 238U/235U isotope ratios in modern carbonates from the Bahamas: Assessing a novel paleoredox proxy. *Chemical Geology* **362**, 305–316 (2013).
6. Herrmann, A. D., Gordon, G. W. & Anbar, A. D. Uranium isotope variations in a dolomitized Jurassic carbonate platform (Tithonian; Franconian Alb, Southern Germany). *Chemical Geology* **497**, 41–53 (2018).
7. Bábek, O. *et al.* Redox geochemistry of the red 'orthoceratite limestone' of Baltoscandia: Possible linkage to mid-Ordovician palaeoceanographic changes. *Sedimentary Geology* **420**, (2021).
8. Tissot, F. L. H. *et al.* Controls of eustasy and diagenesis on the 238U/235U of carbonates and evolution of the seawater (234U/238U) during the last 1.4 Myr. *Geochimica et Cosmochimica Acta* **242**, 233–265 (2018).
